# Cases Treated with Kreosote

**Published:** 1862-01

**Authors:** H. Vantyle Passage

**Affiliations:** Peru, Indiana


### CASES TREATED WITH *KREOSOTE.*

By H. VANTYLE PASSAGE, M. D.,

*Of Peru, Indiana.*

### *Case First.*—Lupus Exedens.

About the 5th of last March, I was called to see Mr. H——, aged 30 years. He informed me that he had a cancer on his nose, of three years standing; that he had employed the services of a number of doctors, and all had assured him it was a cancer. I questioned him in regard to its origin and progress, and ascertained that swelling and inflammation of the *alæ nasi* was the first symptom he noticed of the disease, and it had progressed gradually ever since. At the time of my visit, there was excoriation of the external surface as far up as the arch of the nose, which was partly covered with dry scabs, and the remaining portion of the surface secreting an ichorous matter. The internal surface was in a state of excoriation as far up as the upper portion of the nasar arch (the septum of the nose being destroyed up to the vomer), and secreting a matter much like the external exudation. My diagnosis was, *Lupus Exedens*, or *noli me tangere.*

I adopted the treatment taught me by Prof. Brainard, which was Iodide of Potasii internal, and directed the surface to be washed every day with warm water and *Sapo Cast*, followed by the application of Kreosote. I directed the application to be made with a camel hair pencil and over all the exuding surface.

This treatment was kept up until the cure was completed, which was done in about two months, and there has been no symptom of a return of the disease up to the present (December 10th) time. I give the facts and leave any further comment to the reader.

*Case Second.*—HÆMORRHOIDS.

Rev. Mr. B—— applied to me about the first of June, and informed me that he had been troubled with piles for nine years, and was so troubled at times that he could not ride on horseback. He had three tumors in the rectum, the farthest one being about one and-a-half inches from the external anus. Notwithstanding the liability of internal Piles to bleed, they never had troubled him with hæmorrhage. I ordered that the bowels should be kept open with mild apperients and the rectum washed once a day with *Aqua Pura* and *Sapo Cast*; then apply an ointment to the tumors composed of Kreosote and lard, equal parts. This treatment was followed for about three months, when the patient informed me that the tumors were all gone except the one highest up in the rectum, and that was hanging by a string and moving up and down according to the posture he assumed. I severed the cord-like substance that retained the tumor and it was removed. The old gentleman has attended to his business as an itinerant minister without any inconvenience since the cure was completed; and I may add that the only trouble during the treatment was in applying the remedy.

I have since applied the Kreosote ointment to a case of external Piles, of eight months standing, with a good result, the tumor disappearing in about four weeks.

*Case Third.*—CHANCRE.

I was treating a case of soft Chancre or primary Syphilis, which refused to yield to the lunar caustic. The Chancres were three in number and situated on the gland beneath the prepuce. I applied the clear Kreosote with a camel hair pencil. The patient drew the prepuce over the glands, and as far as the Kreosote came in contact with the mucus membrane it formed a blister, but in two days the blister came off in the form of a scab and the Chancres were gone.

I have since used the Kreosote in a case of Chancre on the external portion of the prepuce, and it was entirely destroyed in seven days.

I am using the Kreosote in two cases of more importance than any of those related, and I will report as soon as I obtain the result.

### TREATMENT OF SEA-SICKNESS.

Thomas M. Hocken, M. R. C. S., and formerly of the ship *Great Britain*, uses the following in cases of sea-sickness :—"Dilute hydrochloric acid, two drachms; dilute nitric acid, one drachm; hydrocyanic acid (Scheele's), sixteen minims; sulphate of magnesia, six drachms; water to eight ounces. Two table-spoonfuls to be taken every three or four hours." Previous to its administration he gives, as he says in the *Lancet*, "a sound purge of calomel and colocynth, or of croton oil on sugar, taken with as little water as possible. Diet, gruel, sago, or arrowroot, with a little brandy, and dry toast and tea.

